# Distinct eye movement patterns enhance dynamic visual acuity

**DOI:** 10.1371/journal.pone.0172061

**Published:** 2017-02-10

**Authors:** Dimitrios J. Palidis, Pearson A. Wyder-Hodge, Jolande Fooken, Miriam Spering

**Affiliations:** 1 Dept. Ophthalmology & Visual Sciences, University of British Columbia, Vancouver, British Columbia, Canada; 2 Graduate Program in Neuroscience, University of British Columbia, Vancouver, British Columbia, Canada; 3 Center for Brain Health, University of British Columbia, Vancouver, British Columbia, Canada; 4 Institute for Computing, Information and Cognitive Systems, University of British Columbia, Vancouver, British Columbia, Canada; 5 International Collaboration on Repair Discoveries, University of British Columbia, Vancouver, British Columbia, Canada; Monash University, AUSTRALIA

## Abstract

Dynamic visual acuity (DVA) is the ability to resolve fine spatial detail in dynamic objects during head fixation, or in static objects during head or body rotation. This ability is important for many activities such as ball sports, and a close relation has been shown between DVA and sports expertise. DVA tasks involve eye movements, yet, it is unclear which aspects of eye movements contribute to successful performance. Here we examined the relation between DVA and the kinematics of smooth pursuit and saccadic eye movements in a cohort of 23 varsity baseball players. In a computerized dynamic-object DVA test, observers reported the location of the gap in a small Landolt-C ring moving at various speeds while eye movements were recorded. Smooth pursuit kinematics—eye latency, acceleration, velocity gain, position error—and the direction and amplitude of saccadic eye movements were linked to perceptual performance. Results reveal that distinct eye movement patterns—minimizing eye position error, tracking smoothly, and inhibiting reverse saccades—were related to dynamic visual acuity. The close link between eye movement quality and DVA performance has important implications for the development of perceptual training programs to improve DVA.

## Introduction

Dynamic visual acuity (DVA) is the ability to resolve fine spatial detail in an object that moves relative to the observer. It is critical for sports performance involving visuo-motor action with fast moving objects. In baseball, for example, a batter must extract information about the moving ball’s motion angle, speed and spin. A close relation has been shown between DVA and level of expertise in various ball sports such as baseball, basketball, volleyball, table tennis, tennis, soccer and water polo [1–9]. These studies show that athletes outperform non-athletes, and that experts exhibit better DVA than novices. Baseball players also show superior static visual acuity [10]. However, it is unclear which factors lead to such DVA advantages.

**Fig 1:**
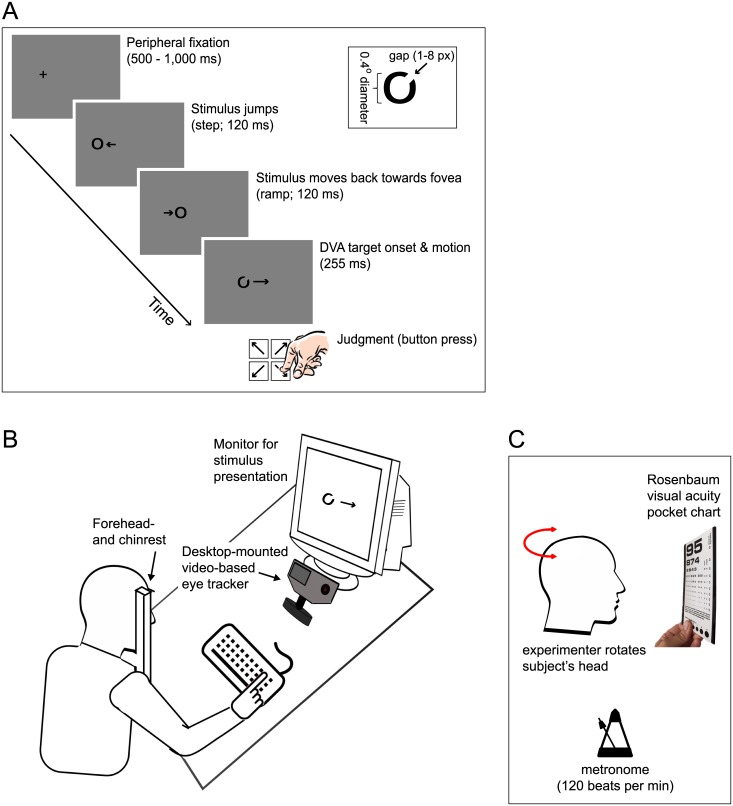
Experimental timeline and set-up. A. Trial timeline of computer-based DVA test. Each trial starts with peripheral fixation, followed by step-ramp stimulus motion; target is a Landolt-C ring. Observers performed a judgment about the location of the gap in the “C” (one of 4 locations) at the end of each trial via button press. B. Set-up for computerized dynamic-object DVA test and eye tracking. C. Set-up for clinical static-object DVA test.

DVA is typically tested in two different ways: by asking observers to identify or judge dynamic objects while their head is fixed (“dynamic-object DVA”), or static objects while their head or body moves (“static-object DVA”). A classic test of dynamic-object DVA involves reporting the location of a small opening in a moving “Landolt-C” ring (see Fig 1a). This type of task measures minimum resolvable acuity, i.e., the ability to separate two features in space. It requires a combination of different types of eye and head movements to stabilize the image of interest close to the fovea, the small area on each eye’s retina where photoreceptor density and visual acuity are maximal [11,12]. Smooth pursuit eye movements aim to match the speed of gaze with that of small, moving visual targets, and can be used to track objects travelling at speeds of up to ~50 degrees of visual angle per second. At higher target speeds, when gaze lags behind the target, the eyes use fast catch-up saccades to compensate for position and velocity errors [13]. A classic static-object test involves recognizing stationary letters or numbers on a visual acuity chart while the observer’s head is being rotated. This test is widely used clinically [14] and requires use of the vestibulo-ocular reflex (VOR), an eye movement induced by activation of the vestibular system. The VOR compensates for head rotation to keep stationary objects of interest close to the fovea.

Eye movements and visual perception are closely related [15,16]. We reason that superior DVA could be attributed to better eye movement control. For example, more effective smooth pursuit enables the viewer to keep a moving object closer to the fovea, thus reducing motion blur [17,18], and pursuit enhances motion prediction [19,20]. In general, image position relative to the fovea and retinal image motion—two factors modulated by pursuit—predict DVA [21,22]. Indeed, recent studies in baseball players have linked better dynamic-object DVA to eye movements [7,8]. However, these studies did not investigate detailed pursuit kinematics or catch-up saccade properties in relation to DVA. Catch-up saccades seem an important factor, given that the speeds of most balls in sports are too high to be tracked smoothly. It is thus unknown which aspects of the eye movement response affect DVA. Here we test DVA in varsity baseball players and identify eye movement patterns that relate to DVA performance. The current study also compares performance in a static-object and dynamic-object DVA test to assess the relation between both types of tasks.

## Methods

### Observers

Participants were 23 males (mean age 19.5 yrs, SD = 1.2), members of the varsity baseball team at the University of British Columbia (UBC), Vancouver, Canada. The research described here was conducted according to the principles expressed in the Declaration of Helsinki, and the UBC Behavioral Research Ethics Board approved experimental protocols. All participants gave written informed consent prior to participation. All participants had normal or corrected-to-normal visual acuity; those with refractive errors wore their regular glasses or contact lenses during the study. Normal visual acuity was confirmed using the ETDRS visual acuity chart at 4-meter test distance (Original Series Chart “R”, Precision Vision, La Salle, IL, USA). Average visual acuity was 20/15 and observers’ acuity ranged from 20/35 to 20/12.5 monocularly, and 20/20 to 20/10 binocularly.

### Stimuli and set-up

We developed a computer-based dynamic-object DVA task in which stimuli were black Landolt-C rings (Fig 1a; luminance 1.1 candela per square meter; cd/m^2^), with a gap in one of four locations, either top right (as shown in Fig 1a), top left, bottom left, or bottom right. Stimulus diameter was 0.40 deg, gap size varied between 1–8 pixels from trial to trial. In each trial, the target moved horizontally to the left or to the right at a constant speed of 50 or 70 degrees per second (deg/s). Target motion direction was randomized from trial to trial. The fixation target was a black cross of 0.40 deg diameter presented 5 deg to the left or right of screen centre. Stimuli were presented on a white background with a luminance of 107 cd/m^2^. Participants were seated in front of a computer monitor (Fig 1b; 18-inch CRT, 40.6 × 30.5 cm, 1600 × 1200 pixels, refresh 85 Hz; ViewSonic, Walnut, CA, USA) at a viewing distance of 71.5 cm. With this setup, 1 pixel is equivalent to 0.0204 degrees of visual angle. Stimulus display and data collection were controlled by a PC (NVIDIA GeForce GT 430 graphics card) and the experiment was programmed in Matlab 7.8 (The MathWorks Inc., Natick, MA, USA) using Psychtoolbox 3.0.8 [23]. A combined chin- and forehead rest stabilized participants’ head.

### Task and procedure

Each trial started with a fixation cross (Fig 1a), and observers were instructed to keep their eyes on the cross until the moving target appeared. To prevent anticipatory eye movements, the fixation cross was displayed for a variable time interval (500–1000 ms). Target motion began simultaneously with the offset of the cross. We used a “step-ramp task”–a paradigm widely employed in laboratory pursuit studies [24]. Because smooth pursuit typically occurs with a latency of 100–150 ms in humans, the sudden appearance of a moving target would cause the eye to jump (saccade) to the target and then track it smoothly. In order to produce a smooth onset of pursuit, the target (a closed circle) initially jumped away from fixation (step) and then moved back towards the fovea, akin to the path of a baseball in the pitcher’s hand. The target reached the fovea after 120 ms, just in time for the eye to start tracking it, and changed from a closed circle to an open Landolt-C ring (Fig 1a). It then continued to move at a constant speed for 255 ms. Observers were instructed to track the Landolt-C ring with their eyes as soon as it appeared, and to report the location of the gap by pressing one of four buttons on the computer keyboard corresponding to the four possible gap locations (4-alternative forced choice procedure; 25% chance of guessing correctly). To assess DVA, we manipulated the size of the Landolt-C’s gap in each trial in a procedure that was adaptive to each individual’s performance (“staircase” procedure). In the first trial, the gap width was set to 7 pixels to make the gap easily visible to each observer. In subsequent trials, the gap width was reduced if observers gave a correct response three times in a row, and increased if observers made a mistake (1-up-3-down adaptive procedure; [25]). Gap width was modulated in 2-pixel increments until the first reversal point (going from “correct” to “incorrect” or vice versa), and in 1-pixel increments thereafter. Separate staircases were run for the two speed conditions with conditions randomly interleaved in blocks of 80 trials maximum. On average, each observer completed 116 trials, and the experiment took a maximum of 30 minutes. Perceptual thresholds were then determined for each staircase as the mean gap width at all reversal points, excluding the first reversal.

Following computerized assessment of dynamic-object DVA, we next used a standard clinical test to measure static-object DVA. Observers were seated in a chair with the experimenter standing behind them and viewed a visual-acuity pocket eye chart (Rosenbaum pocket vision screener; Prestige Medical Inc., Northridge, CA) positioned in front of them at a distance of 35 cm. Participants were instructed to read aloud the numbers on the acuity chart from top (large font; 20/800 vision) to bottom (small font, 20/20 vision) during head rotation. This is a well-established procedure frequently used in clinical settings. To reduce variability we introduced additional experimenter control over the task by having the experimenter actively rotate the observer’s head gently to the beat of a metronome (120 beats per minute; Fig 1c).

### Visual acuity conversions

Static visual acuity (ETDRS chart) and static-object DVA (Rosenbaum chart) were recorded as Snellen acuity and converted to degrees of visual angle following conversion tables [26]. Normal vision, i.e., 20/20 Snellen acuity, corresponds to a LogMAR score of 0, equivalent to 1 MAR or 0.0167 degrees. To calculate the acuity of observers who resolved all but one or two of the symbols on their last attempted line of the ETDRS or Rosenbaum visual acuity charts, each letter missed was weighted as a fraction of the numbers or letters on that line. For example, the ETDRS chart has five letters per line. If the observer resolved all letters on the line corresponding to 20/25 Snellen acuity (0.0208 degrees) but failed to read one of the symbols on the line corresponding to 20/20 Snellen acuity (0.0167 degrees), then that observer would be evaluated as being able to resolve a visual angle of 0.0175 degrees ([(0.0208–0.0167)/5] + 0.0167).

### Eye movement recording and preprocessing

During computer-based testing, position of the right eye was recorded with a desktop-mounted video-based eye tracker (Fig 1b; Eyelink 1000 Desktop Mount; SR Research Ltd., Ottawa, ON, Canada) and sampled at 1000 Hz, i.e., yielding one sample of eye position data per millisecond. Eye movements were analyzed off-line using custom-made routines in Matlab [19]. Eye position, velocity, and acceleration profiles were filtered (smoothed) and saccades were detected when three consecutive samples exceeded a fixed velocity criterion of 75 deg/s. Saccade on- and offsets were determined as the nearest zero crossing of the acceleration occurring before and after the samples exceeding the velocity criterion, respectively. Visual inspection was used to verify saccade detection. If saccade onset/offsets did not coincide exactly with the closest acceleration minimum and maximum, respectively, manual correction was used to correct for small detection errors. We then segmented the remaining eye movement traces into fixation and smooth pursuit based on the eye trajectory’s angular dispersion, using directional statistics to determine whether the eye moves in a consistent direction, as in smooth pursuit.

For each trial, we computed the following eye movement parameters as indicators of observers’ eye movement quality: (a) Smooth pursuit latency, the temporal differences between DVA target onset and onset of the smooth part of the eye movement. The analysis of latency was only based on trials with a smooth pursuit onset in which tracking was initiated between 100 ms after stimulus onset (to exclude trials with anticipatory pursuit) to stimulus offset. (b) Smooth pursuit eye position error, the absolute difference between the horizontal position of the eye and the stimulus, computed for the time period between DVA target onset and offset, during the non-saccadic portion of the trace. (c) We also calculated the minimum position error occurring in this time interval as indicator of the closest distance to the target achieved by the eye. (d) Smooth pursuit gain, the saccade-free eye velocity divided by target velocity in the final 200 ms of stimulus presentation for trials in which tracking onset had already occurred prior to this point—either in the form of a saccade or smooth pursuit onset. (e) Cumulative saccade amplitude, the sum of the amplitude of all saccades that occurred between target onset and offset. (f) Direction of the first saccade, specifically, the proportion of trials in which the first saccade after stimulus onset went in the direction of the stimulus step (reverse saccade). Because the stimulus stepped away from the fixation position and then moved back towards it for the first 120 ms before passing it (the pre-ramp period), subjects could employ two distinct strategies: they could either initiate tracking in the direction of stimulus motion, using the pre-ramp period to offset the cost of eye movement latency and initial acceleration, or they could initiate a saccade in the opposite direction of stimulus motion in an attempt to intercept the stimulus before it reached the fixation position.

We excluded trials from further analysis when data were lost due to blinks during stimulus presentation or lost signal from the eye tracker (82 trials total; approx. 3.5% across all subjects and conditions). Observers for whom we were unable to obtain clear cornea reflection due to reflections off their eye glasses or contact lenses were not included in the eye movement analysis (subjects #8 and #57), yielding usable eye movement data sets from 21 observers.

### Statistical analysis

Main effects of target speed on DVA and oculomotor performance were evaluated using repeated-measures ANOVA. To assess any potential effects of baseball playing experience, we added seniority as a between-subjects factor; n = 11 were junior (freshmen, yr 1) and n = 12 were senior players (yrs 2–4). To investigate the link between eye movement quality and DVA, we used correlational analyses, reporting bivariate correlation coefficients and results of two-sided significance testing at a level of α = 0.05. For all tests we report effect sizes, either r, ƞ^2^, or Cohen’s d. All statistical analyses were conducted in IBM SPSS Statistics Version 24 (Armonk, NY, USA).

## Results

The main purpose of this study was to link individual eye movement kinematics to DVA performance. We will report results in three steps: first, we will describe perceptual DVA and relate dynamic-object DVA to static visual acuity and clinical-type static-object DVA. These results are shown separately for junior and senior players, because effects of expertise have been reported throughout the literature. Second, we will describe oculomotor performance in our computerized dynamic-object DVA paradigm. Third, we will identify eye movement characteristics that are related to dynamic-object DVA. Seniority was not a significant factor driving eye movement kinematics, and these results were thus averaged across all players.

### Dynamic visual acuity in dynamic- and static-object tasks

**Fig 2:**
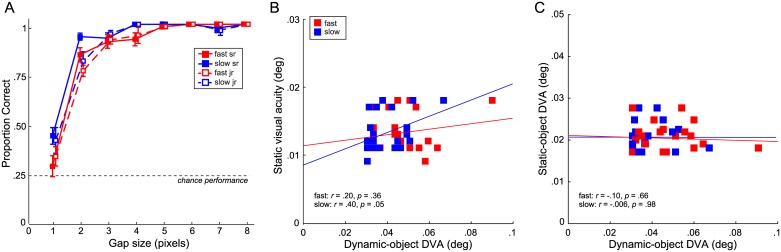
Perceptual acuity results. A. Proportion correct in dynamic visual acuity task as a function of gap size for n = 23 observers; n = 11 were junior players (open symbols) and n = 12 were senior players (filled symbols). Speed is denoted by color, seniority by line type; error bars are standard errors of the mean. B. Relation between static visual acuity (binocular ETDRS) and dynamic-object DVA at slow and fast speed. Lines are best fit linear regressions. C. Relation between static-object and dynamic-object DVA in degrees of visual angle.

Fig 2a shows proportion of correct trials as a function of gap size for two speeds in the computer-based DVA task. We found significantly better performance for slower than for faster speeds with average thresholds at 1.95 and 2.31 pixels, respectively, corresponding to .040 and .047 degrees of visual angle (main effect of speed, F(1,21) = 8.80, p = .007, ƞ^2^ = .30). Senior players—team members with >1 year collegiate experience—had lower thresholds (.037 for slow and .044 for fast) than junior players (.043 and .050, respectively; compare solid and dashed lines in Fig 2a). However, thresholds were not significantly different (no main effect of between-subjects factor seniority, F(1,21) = 2.43, p = .13, ƞ^2^ = .14). For both speeds and both groups, performance rose quickly going from near chance at 1 pixel gap size to >80% correct at 2 pixels. Performance saturated to near perfect at 4 pixels for the slow speed and 5 pixels for the fast speed, indicating low rates of attentional lapse.

All participants completed standard visual acuity testing using an ETDRS letter chart. Interestingly, dynamic-object DVA performance was related to general visual acuity at slow target speed (r = .40, p = .05; Fig 2b), but not at fast speed (r = .20, p = .36). This finding indicates that visual acuity might contribute to slow-target DVA, but might not limit perceptual performance in response to fast targets.

We assessed DVA in two ways—during head fixation and during head rotation, using dynamic vs. static objects, respectively. We found no correlation between computer-based assessments of DVA with dynamic objects during head fixation vs. clinical examination of DVA with static objects during head rotation (Fig 2c), indicating that both tests assess different aspects of DVA, in accordance with the different types of eye movements they require. Similar to dynamic-objects DVA, seniority also had no effect on static-object DVA (independent-samples t-test, t(21) < 1, n.s.).

### Eye movements during the dynamic-object DVA task

**Fig 3:**
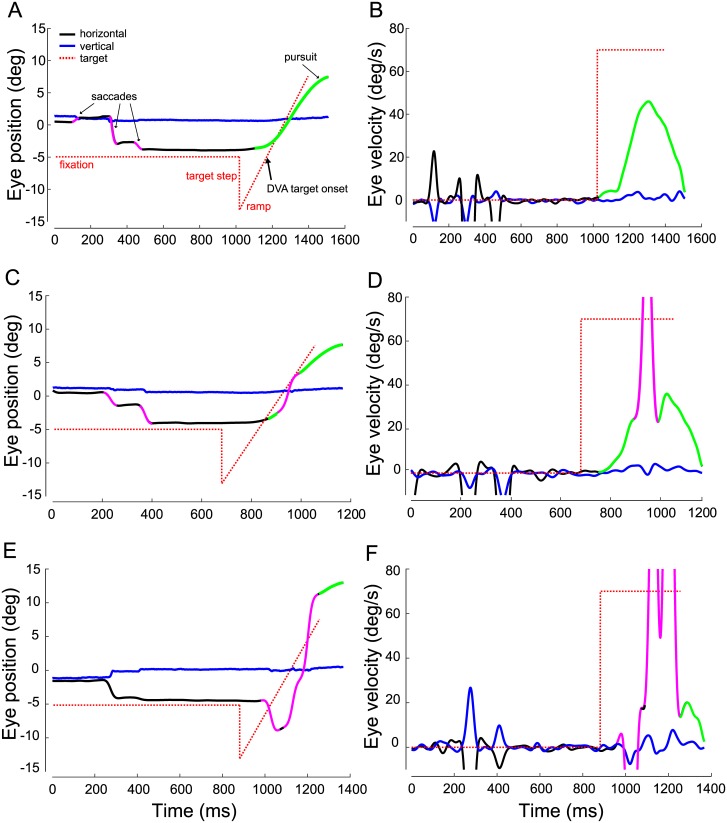
Eye movement responses in dynamic-object DVA task. Eye position (left column) and eye velocity traces (right column) as a function of time from three different trials for a stimulus moving at 70 deg/s; all traces obtained from representative observer #16. Dotted red line denotes target trajectory: peripheral fixation at 5 deg from screen center, target step, target ramp, DVA stimulus onset at time when stimulus reaches fovea, 120 ms after ramp onset. Horizontal eye position in black, vertical eye position in blue. Smooth components marked in green, saccades marked in magenta. A,B. Trial with smooth pursuit (green) following target onset. C,D. Trial with a large, early catch-up saccade (magenta) following target onset. E,F. Trial with a reverse saccade towards the target step, i.e., in the opposite direction to target motion.

To address the question what determines DVA performance across speeds, we next assessed smooth pursuit responses to DVA targets. Fig 3 shows representative eye position and velocity traces in response to faster stimulus motion and reveals different eye movement patterns. In some trials, observers were able to lock their eyes onto the target promptly after it reached the fovea, or even before, and to then track it smoothly (Fig 3a). However, the eye did not reach target velocity (Fig 3b). In other trials, after a brief period of smooth pursuit, observers caught up with the target by making a large saccade (Fig 3c and 3d). In about 30% of all trials across observers, we found that players tried to intercept the target early by making a saccade in the opposite direction of stimulus motion (reverse saccade) before changing the direction of eye movement (Fig 3e and 3f). This pattern occurred frequently even though the step-ramp stimulus design was employed to facilitate smooth pursuit initiation in the direction of the target. The use of reverse saccades was suboptimal and often resulted in less effective tracking, i.e., no pursuit or delayed pursuit onset (Fig 3e and 3f).

Consistent with perceptual results, smooth pursuit eye movements were faster, smoother, and more accurate when tracking slow as compared to fast target motion. However, pursuit onset, minimum position error, and reverse saccades were unaffected by speed (Table 1). We found no main effect of seniority on any of the pursuit measures (all p > .17), but junior players made reverse saccades in almost twice as many trials (42%, SD = 23.3) as compared to senior players (23%, SD = 16.6). When analyzed separately by speed, this difference was significant for slow speed and revealed large effects (t(19) = 2.13, p = .046, d = .92), but not significant for fast speed (t(19) = 2.05, p = .05, d = .89).

**Table 1:** Effects of speed on smooth pursuit eye movements. Shown are means and standard deviations for slow and fast speed and results of repeated-measures ANOVA with speed as factor.

	Mean (SD) slow	Mean (SD) fast	F(1,19)	p	ƞ^2^
**Latency**	148.9 (16.5)	146.6 (16.6)	.72	.41	.05
**Abs. position error**	1.77 (.46)	2.18 (.86)	5.86	.03	.28
**Min. position error**	.60 (.38)	.53 (.36)	3.68	.07	.20
**Gain**	.68 (.19)	.51 (.17)	115.29	.001	.89
**Saccade amplitude**	7.9 (3.6)	10.5 (4.8)	21.17	.001	.59
**Reverse saccades**	.32 (.24)	.32 (.19)	0.07	.93	.00

### Is perceptual performance linked to smooth pursuit?

**Fig 4:**
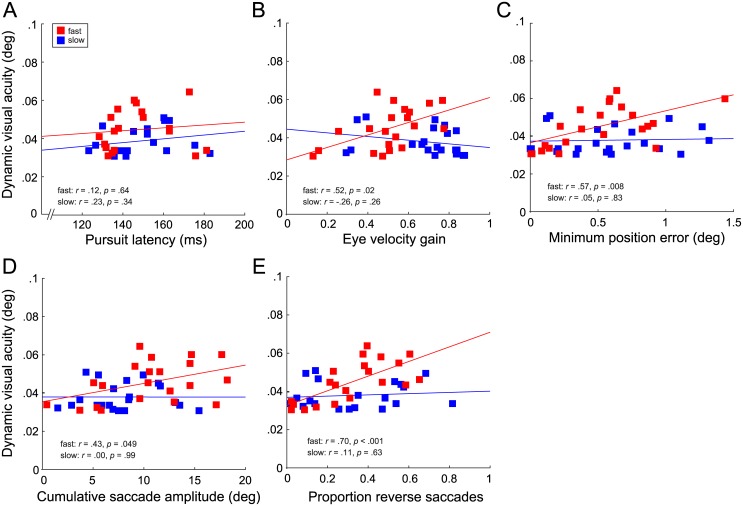
Correlations between perceptual DVA thresholds and eye movement parameters. Dynamic-object DVA thresholds are given in degrees of visual angle; results are for n = 21 observers with available eye movement data. Each data point is one observer in a given category; speed is denoted by color. Lines denote best linear fits. A. Eye velocity latency (ms). B. Eye velocity gain. C. Minimum position error (deg). D. Cumulative saccade amplitude (deg). E. Proportion of trials with reverse saccade.

To identify which aspects of eye movement kinematics were related to perceptual performance, we correlated eye movement metrics—latency, velocity gain, absolute and minimum position errors, cumulative saccade amplitude, and proportion of trials with a reverse saccade—with perceptual thresholds on an individual observer basis. At slow target speed, there was no relation between any of the eye movement metrics and perceptual performance (Fig 4). However, at fast target speed, where eye movements are likely a limiting factor to performance—humans cannot usually track targets moving at 70 deg/s smoothly—we found a strong relation between all metrics and DVA, except latency (Fig 4a). Better perceptual performance, i.e., a lower DVA threshold, was associated with a smaller minimum eye position error (Fig 4c). Thus, observers who were better able to center their gaze on the target at any time during the trial were better able to resolve fine spatial detail. Reverse saccades, likely made to intercept the target before it reached fixation (see Fig 3e and 3f), showed the strongest relation with perceptual performance (Fig 4e): a higher proportion of trials with reverse saccades was associated with higher DVA thresholds. Similarly, subjects who covered more overall distance with saccades performed more poorly in the perceptual task (Fig 4d). This relationship might be mediated by reverse saccades, associated with poor DVA, and causing an increase in cumulative saccade amplitude. In support of this interpretation, the proportion of reverse saccades was highly correlated with cumulative saccade amplitude (r = .67, p < .001). The partial correlation between cumulative saccade amplitude and DVA was non-significant when controlling for proportion of reverse saccades (r = -.07, p = .79). In conjunction with the finding that senior players made fewer reverse saccades than junior players this result indicates that athletic experience might be linked to the use of more effective oculomotor control decisions. In this particular task, with the chosen target speeds, an effective oculomotor strategy is characterized by an efficient and accurate use of saccades in the direction of the pursuit target.

Interestingly, better perceptual performance was related to a lower eye velocity gain at fast target speed (Fig 4b), confirmed by a significant positive correlation between gain and DVA threshold (r = .52, p = .02), i.e., high gain is associated with a high DVA threshold and thus with poor performance in this task. This finding indicates that observers who attempted to match the speed of their eye movement to that of the target by using smooth eye movements alone performed more poorly. In line with this result, we found a highly significant positive correlation between gain and minimum position error at fast speed (r = .65, p = .001), suggesting a tradeoff between high pursuit gain and low minimum position error. Indeed, the relation between gain and DVA is no longer significant when controlling for minimum position error (r = .24, p = .32).

Overall, the correlations between perceptual performance and eye movement metrics indicate a stronger relation between DVA and saccade parameters—reverse saccades, cumulative saccade amplitude and minimum position error, which can be achieved by making a saccade—than with parameters reflecting high-quality smooth tracking.

## Discussion

This study shows that dynamic visual acuity is related to two distinct eye movement metrics: minimum position error and the frequency of reverse saccades. Minimum position error corresponds to how well gaze is aligned with a moving object of interest, such as a baseball. The occurrence of reverse saccades likely reflects a failure to produce or learn optimal saccade control. We also found that static visual acuity was only related to dynamic-object DVA at slow speed, indicating that DVA performance at fast speed is not constrained by visual acuity alone. Static-object DVA and dynamic-object DVA were unrelated and can thus be considered tasks that measure different aspects of DVA.

Previous studies investigating DVA consistently report large effects of expertise. Our study did not reveal such differences between junior and senior baseball players. The cohort of varsity athletes tested here might have been more homogenous than cohorts of athletes and non-athletes, or experienced and naïve athletes that have been compared in previous studies [1–9].

### Eye movements and dynamic visual acuity

Our study identifies specific eye movement metrics as potential predictors of DVA. Previous studies have not quantitatively differentiated the role of different types of eye movements, particularly smooth pursuit and saccades, in their contribution to DVA. This is surprising, given the importance of these types of eye movements in ball tracking in the real world [27,28] or in a virtual-reality sports setting [29]. Uchida et al. compared DVA during fixation and pursuit in response to stimuli traveling at very high speeds (200–900 deg/s) and found improved DVA when observers were allowed to track the target with their eyes vs. when they were asked to fixate [7]. Baseball athletes performed better than non-athletes in the free eye movement condition but not during fixation, indicating that superior DVA in athletes is due to better tracking, not image processing. In their 2013-study, the same authors attribute superior DVA in athletes to eye movements with shorter latency, higher peak velocity, and lower minimum position error [8]. However, the stimulus velocities in this study were beyond the limits of human smooth pursuit [30] and indeed Uchida et al. [7,8] did not specify whether they observed smooth pursuit. Their paradigm did not involve a step-ramp procedure to smoothly initiate pursuit, and participants likely used saccades to reduce the initially accumulating position error, thereby matching eye velocity to stimulus velocity at some point during the negative acceleration phase following the saccade. The current study used much lower stimulus velocities and employed a step-ramp procedure to initiate pursuit.

Interestingly, high smooth pursuit velocity gain in our study was associated with poorer perceptual performance at fast target speed, indicating that observers who attempted to match the speed of their eye movement to that of the target, using smooth pursuit, performed more poorly. We hypothesize that this result reflects a tradeoff between achieving high pursuit gain and low minimum position error, which was supported by a positive correlation between the two metrics at high target speed. The observed increase in position error might be the cost of continuously matching eye velocity to high target velocity.

It seems that observers frequently used saccades to rapidly and effectively reduce position error (see Fig 3). In general, high velocity gain is necessary to match target speed and to reduce motion blur [17,18]. High-quality pursuit is also beneficial for tasks in which observers have to make predictions about a target’s trajectory [19,20]. However, our DVA task confronted observers with targets moving at high speeds along a predictable trajectory. Observers had to discriminate the location of a small target feature, requiring them to bring the target into the fovea just long enough to do so. This can be achieved by matching the speed of the eyes to that of the target during the negative acceleration phase of a catch-up saccade. Because position error is typically at a minimum at the end of an accurate catch-up saccade, there is a brief moment of low position error and low retinal slip, during which the target could be perceived at high acuity. Because this occurs during the trajectory of a saccade, it is not reflected in our measure of smooth pursuit gain. This saccadic strategy likely also underlies the findings reported by Uchida et al. [8], although their stimulus velocities made smooth pursuit impossible, while in our paradigm pursuit was often achieved but possibly not effective. Overall, it seems that the use of high-gain smooth pursuit is less critical to DVA (see Fig 4b) than minimizing position error (Fig 4c), especially if this is achieved with an optimal saccade strategy (Fig 4d and 4e). Frequent and high-amplitude saccades are equally detrimental to DVA performance (Fig 4d) as the attempt to match target speed with smooth pursuit alone.

Moreover, we found that the use of a reverse saccade to intercept the target before it reached the fixation point was associated with poor perceptual performance, indicating a suboptimal strategy. With a slower target this maneuver might lead to a more immediate reduction in position error and more time with the eye on the target. However, depending on task constraints, it potentially introduces increased error if the player is unable to accurately judge the position and trajectory of the target, or to plan and execute the saccade and subsequent reversal of eye movement direction. Players achieved higher DVA by not attempting to correct for the initial position error, in accordance with the optimal feedback control strategy of minimum intervention, in which errors are not corrected unless they interfere with task performance [31]. This finding could reflect superior ability to use and adapt internal models of the target state and the control of the eye. Such forward models are used to predict the sensory consequences of movements [32]. Past research has shown that ball sport athletes demonstrate a faster rate of saccadic adaptation to experimentally imposed position errors [33]; a process purported to result from updating of internal forward models [34]. We observed that more experienced players performed the suboptimal strategy of using reverse saccades significantly less frequently at the higher target speed.

In general, oculomotor predictors of DVA likely depend on the parameters of the task. We used a task with stimuli presented briefly and moving at speeds near the limits of smooth pursuit to mimic real-world task requirements in baseball.

### Dynamic-object versus static-object DVA

DVA can be tested either by asking an observer to identify static objects during head rotation, or to judge dynamic objects while the observer’s head is fixed. These two types of DVA-tests require different types of oculomotor control mechanisms: the VOR compensates for fast head rotation, whereas smooth pursuit eye movements serve to keep small moving targets close to the fovea when the head is relatively stable. Thus, not surprisingly, these tests examine different aspects of DVA, which are uncorrelated in our study. In principle, both types of eye movements are needed to successfully stabilize the image of a baseball on the retina, especially in outfielders who have to track the ball with their eyes while their body is in motion. Even though hitters naturally rotate their upper torso and head when they hit the ball, little head rotation is needed during trajectory estimation. The VOR is suppressed during head rotations in professional batters [27]. Rather, tracking a pitched baseball requires a combination of smooth pursuit, saccades, and vergence eye movements to focus on the ball moving through different depth planes as well as anticipatory saccades [28].

## Conclusion

In recent years, visual and perceptual components have become a core component in athlete performance assessment [35–38]. Our study reveals the importance of smooth pursuit eye movements for the ability to resolve spatial detail in moving objects and identifies patterns that might enhance perceptual performance. Eye movement tasks could be useful additions to perceptual training programs for baseball [39] and might potentially provide useful tools in assessing and recruiting athletes.

## Supporting information

S1 Dataset Complete set of perceptual and oculomotor data. (ZIP)
