# Supplementary material for: Distinct eye movement patterns enhance dynamic visual acuity
Source: PLoS One. 2017 Feb 10;12(2):e0172061. doi: 10.1371/journal.pone.0172061 (PMC5302791; doi:10.1371/journal.pone.0172061)
Supplement: S1 Dataset — (ZIP) [file pone.0172061.s001.zip › readMe.rtf]

‘PalidisEtal_DVA_Acuity_Batting’ is an Excel document containing information about the perceptual and biographical participant information. The first row labels contains column labels, while each subsequent row corresponds to a participant. Columns correspond to participant ID, Age, whether they are classified as a senior member of the team, their dynamic object DVA threshold at slow and fast speeds, their static object DVA, and their static acuity.‘playerEyeData’ is Matlab struct Array with length=21, with one element per subject with usable eye data. For each element, the field ‘ID’ contains a string with the subject’s ID number, the field ‘eyePositionXY_Degrees’ contains a cell array of size 1xNumber of trials, each containing eye position data in degrees of visual angle sampled at 1000hz- the first column contains horizontal position, the second contains vertical position. The field ‘targetData’ contains a struct array of length= number of trials containing the target motion direction, speed, and onset in milliseconds relative to the start of eye position recording.
